# Laser Interstitial Thermal Therapy for the Treatment of a Pineal Region Glioma Through an Infratentorial Approach: A Case Report

**DOI:** 10.7759/cureus.33607

**Published:** 2023-01-10

**Authors:** Omar Nabulsi, Mohamed Abouelleil, Sanjay Patra, Paul Mazaris

**Affiliations:** 1 Neurosurgery, Spectrum Health Medical Group, Grand Rapids, USA; 2 Neurological Surgery, Spectrum Health Medical Group, Grand Rapids, USA

**Keywords:** pineal region glioma, laser interstitial thermal therapy, excision of tumor, pineal region tumor, infratentorial, pineal region

## Abstract

Laser interstitial thermal therapy (LITT) is a minimally invasive surgical option for the treatment of brain tumors introduced in 1983. The innovative technique was welcomed for its ability to access deep-seated supratentorial and posterior cranial fossa lesions. Surgical approaches to pineal region tumors are challenging and require a high degree of precision since the critical vasculature, such as the vein of Galen and precentral vein, in the area pose significant anatomical challenges to operating surgeons. To minimize the risk of damaging this key venous anatomy, an infratentorial approach may be more advantageous. We present a case where LITT was utilized through an infratentorial approach to a pineal region tumor.

A 62-year-old male with no significant past medical history presented to his primary care physician complaining of ataxia and headaches for the past four weeks. An MRI was concerning for multicentric glioma within the cerebellar hemispheres, brainstem extending to the middle cerebellar peduncle, upper cervical spinal cord, and pineal region. An enhancing lesion of the midbrain tectum was concerning for a high-grade tumor. We decided to proceed with stereotactic biopsy and magnetic resonance-guided LITT via an infratentorial approach. Supratentorial trajectory planning did not allow for a safe corridor due to the venous anatomy; thus, it was decided to proceed with an infratentorial approach. The patient was positioned prone, had his bone fiducial CT fused with MRI, and the tumor was targeted using robotic guidance (ROSA, Zimmer Biomet, Warsaw, Indiana). Postoperatively, he suffered from transient diplopia due to cranial nerve VI palsy. Additionally, the postoperative MRI revealed a decrease in the size of the enhancing lesion and the hyperintense T2 signal within the brainstem.

Open surgical approaches to tumors within the pineal region often pose an anatomic and neurovascular challenge. We describe the safe utilization of a novel, previously unreported infratentorial approach utilizing LITT with promising treatment, morbidity, and efficacy outcomes. A larger series will be necessary to ensure the safety and efficacy of this approach.

## Introduction

Pineal region tumors are rare, making up less than 1% of all primary brain tumors in adults [1]. Pineal tumors can be classified as germ cell tumors, pineal parenchymal tumors, and tumors that derive from adjacent anatomical structures [1]. Germ cell tumors are the most common pineal region tumor while glioblastoma multiforme of the pineal region is the rarest form with only 40 reported cases [1,2].

The standard first-line treatment for most symptomatic non-germ-layer benign and malignant brain tumors is surgical excision [3]. Radiosurgery, while widely used for the treatment of brain metastasis, provides unsatisfactory outcomes for high-grade gliomas [4]. For deep-seated tumors, maximal surgical resection remains a challenge, particularly within the pineal region where the surrounding vascular structures impede access. Since its introduction in 1983 by Brown, laser interstitial thermal therapy (LITT) has emerged as a promising, less invasive option for deep-seated lesions [5,6]. Tumors in both the posterior fossa and the pineal region have typically been resected via an open or endoscopic approach [7].

We report the first application of LITT to the pineal region through an infratentorial approach.

## Case presentation

**Figure 1:**
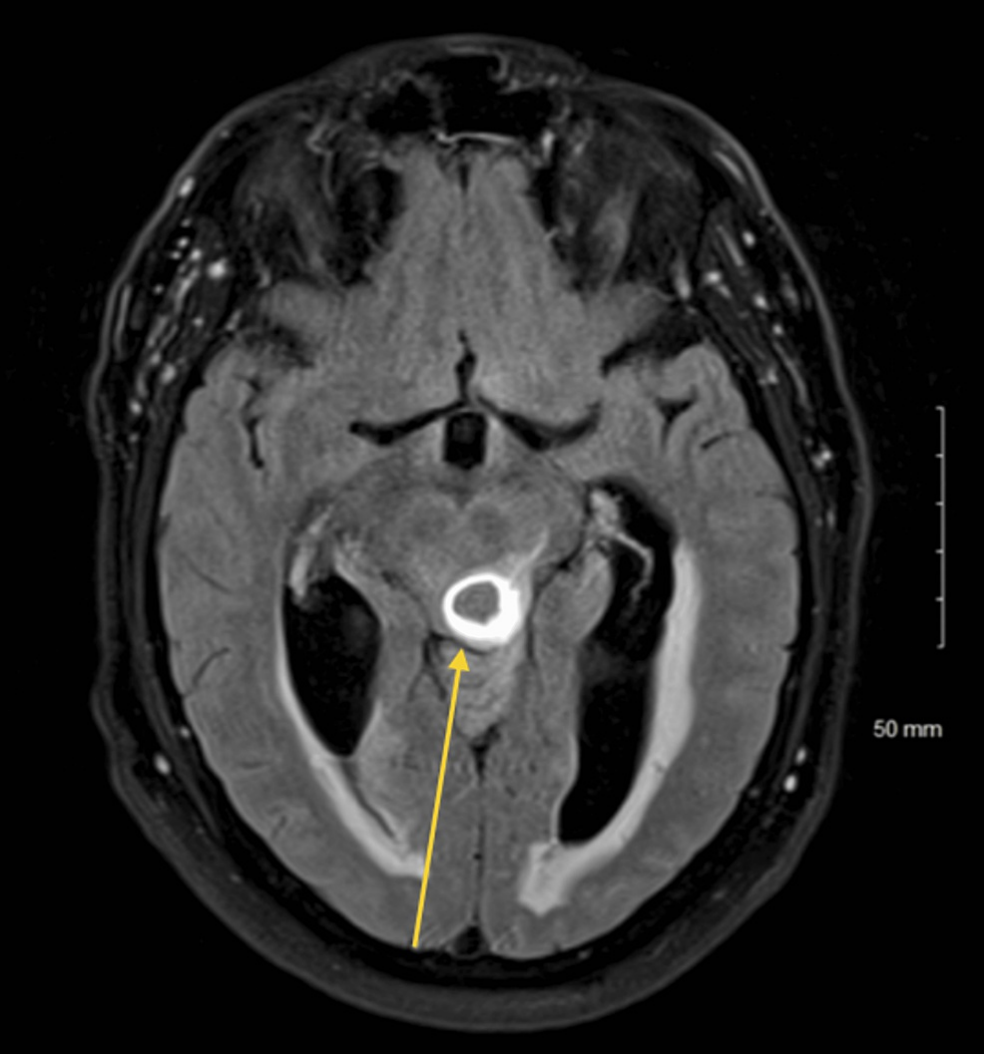
Preoperative MRI Preoperative MRI concerning for multicentric glioma with a dominant lesion involving the brainstem and extending to the left middle cerebellar peduncle greater than the right cerebellar hemisphere and possibly the upper cervical cord.

A 62-year-old male with no significant past medical history presented to an outside hospital complaining of a posterior headache and ataxia for the past three to four weeks. The patient described his headache as 5/10 in intensity and associated with nausea and loss of appetite. The patient was neurologically intact and had no other complaints. An MRI revealed findings concerning for a multicentric glioma, with a dominant lesion involving the brainstem with effacement of the cerebral aqueduct causing obstructive hydrocephalus (Figure 1). The patient was then transferred to our facility for further workup and treatment.

The patient underwent an endoscopic third ventriculostomy. This was not successful in controlling the hydrocephalus; therefore, a right occipital ventriculoperitoneal shunt was placed. Given the multiple regions of T2 hyperintensity, indicating the diffuse nature of the lesion, along with the punctuate contrast enhancement on T1, we decided to perform a biopsy and treat the enhancing component through MRI-guided LITT. Trajectories were initially planned supratentorially; however, due to the configuration of the venous anatomy, a safe approach could not be identified. Therefore, a supracerebellar, infratentorial approach through the cerebellum was used. The patient was placed prone in Mayfield pins. The trajectory was planned on ROSA software (Zimmer Biomet, Warsaw, Indiana), which was used to navigate the trajectory. A two-inch incision was needed to provide enough exposure through the occipital musculature to drill and place the Visualase anchoring bolt. A fiberoptic probe was then placed in the center of the lesion, and the patient was taken to the intraoperative MRI for ablation, receiving energy equivalent to 43°C for two minutes.

**Figure 2:**
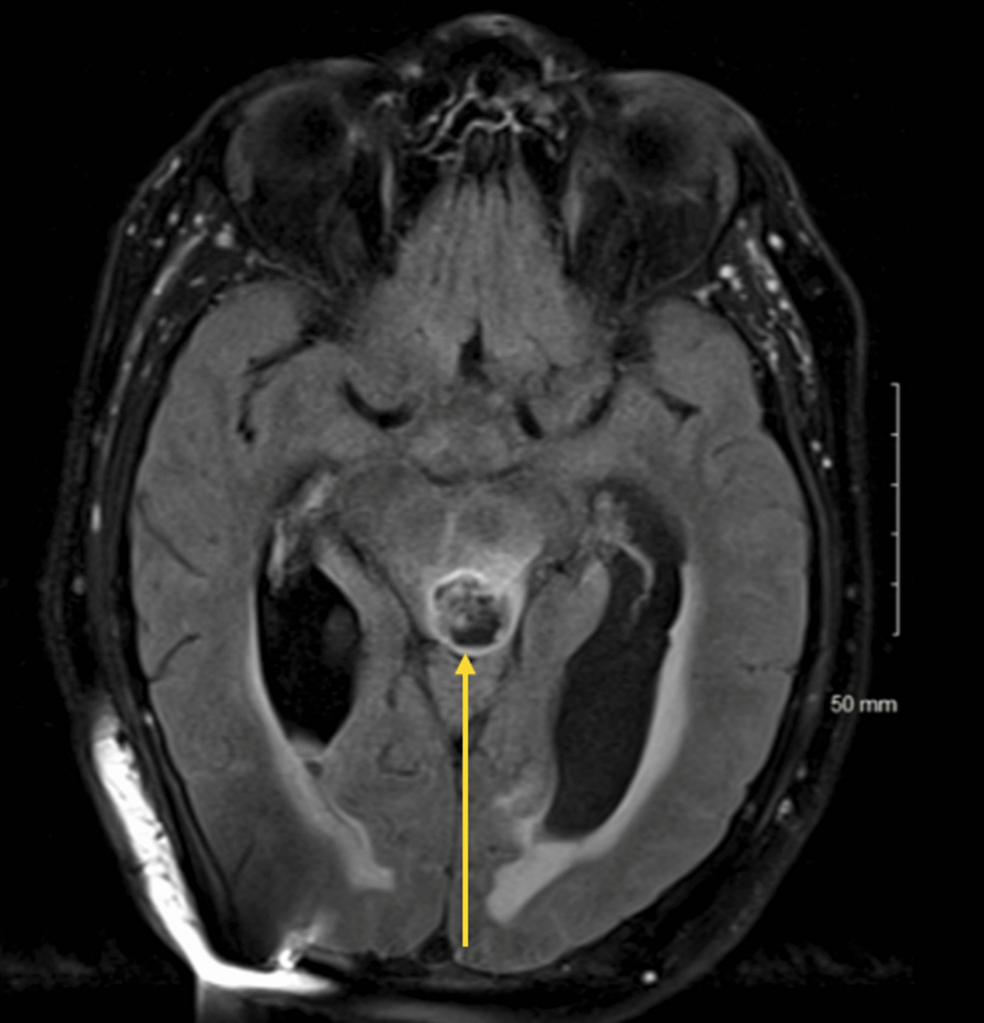
Postoperative MRI Postoperative MRI revealed an interval decrease in the volume of abnormal infiltrative hyperintense T2 signal in the brainstem. The enhancing component of the lesion has also decreased in size, with a further decrease in the size of the ventricles and interstitial edema.

Postoperatively, the patient experienced transient diplopia due to a right cranial nerve IV palsy, and he continued to suffer from ataxia. He underwent radiation therapy and was put on Temodar for chemotherapy. Postoperative MRI at four months showed a decrease in the size of the ventricles and a decrease in the volume of the hyperintense signal and enhancing component of the lesion in the brainstem (Figure 2).

## Discussion

LITT was an innovative technique when it was first introduced and adopted into the surgical arsenal in the fight against tumors in 1983. Despite its novel ability to ablate tumors by heating cells beyond a critical temperature, it was sparingly used in the brain because surgeons were unable to precisely monitor and control the thermal output of the laser probe, which is necessary for the protection of critical brain structures [8]. There have been many improvements in the technique since the 1980s, such as coupling it with real-time MRI thermal imaging, also known as magnetic resonance-guided LITT (MRg-LITT), the development of sophisticated analytical software capable of processing imaging and thermal data and predicting the extent of tissue damage, and general improvements in the design of the laser probe [8]. Advanced MRI-based imaging features, such as diffusion tensor imaging (DTI) and fiber tracking, have allowed for improved intra-operative imaging feedback with LITT, making it far more safe and effective for neurosurgical use [6-9].

Significant improvements in LITT have prompted neurosurgeons to increasingly adopt the technique and report its advantages over conventional surgical options for brain tumors. Compared to more invasive surgery, LITT may be preferred due to its accessibility to hard-to-access lesions and the lesser risk of complications, especially in poor surgical candidates and patients who need prompt initiation of chemotherapy [7-10]. Not only may LITT reduce complications, but it may also increase the efficacy of postoperative chemotherapy due to the disruption of the blood-brain barrier [11]. Lastly, using LITT reduces total hospital stay as it does not typically require patients to recover in the intensive care unit postoperatively; most patients can be discharged home the next day [7-10].

Because of these benefits, LITT should be considered as an alternative to craniotomy if it can provide similar postoperative results with minimal complications. A meta-analysis reported on 829 lesions, consisting of high-grade gliomas, low-grade gliomas, metastatic tumors, and non-glial tumors, and found that the one-year overall survival was 43.0% among high-grade glioma, 45.9% in grade 4 astrocytomas, 93.0% for low-grade gliomas, and 56.3% (47.0-65.3%) in brain metastases, proving to be an effective replacement for other methods [12]. While this meta-analysis focused on the outcomes based on tumor grade, other studies present in the literature concentrate on LITT outcomes based on lesion location. One of which was a review of 58 patients who received LITT for posterior fossa lesions, often difficult to access through craniotomy, which found that 84% of patients did not have any tumor progression at the 9.5-month postoperative follow-up [13]. Another study reviewed 206 patients who received LITT for spinal metastases and reported that all patients had effective local control of the disease with a reduction of epidural compression at 30 days [14]. These findings are encouraging for neurosurgeons who want to begin using LITT in anatomical structures and regions that have not been previously reported on, such as tumors in the pineal region, as was the case for our team.

LITT used specifically for glioblastomas (GBM) treatment is a bit more complex. Because GBMs are high-grade tumors with fast, aggressive growth, LITT may not make a significant difference in their ultimate prognosis when compared to other methods of therapy. A review by Kamath et al. of 58 LITT operations in 54 patients for GBM ablation in both lobar location and deep structures revealed that the median post-LITT survival for patients with recurrent GBM was 11.5 months, of which about 6.6 months were free of tumor progression [15]. This is not much longer than the survival of patients with GBM treated with just bevacizumab chemotherapy at 9.2 months [16]. A cohort study of 20 patients receiving LITT for GBMs solidified this by showing comparable overall survival (11 months) to patients treated with conventional resection [17]. These findings should not discourage the use of LITT in the case that the patient does want to undergo a surgical procedure. In this case, LITT should still be highly considered due to its quicker hospital discharge, faster healing time, and fewer complications. On the other hand, if a patient solely wants chemotherapy, they can rest easy knowing that LITT would not have made much of a difference to their overall survival time.

While these reviews show promise for LITT's application in tumor excision, more research is needed to fully understand the adverse effects of this method. The most notable complications, albeit most transient, after LITT include transient and permanent weakness, cerebral edema, hemorrhage, seizures, hydrocephalus, and hyponatremia [12,15,18]. Although visual deficits are not reported as a common complication of LITT in the context of tumor ablation, they have been reported as a complication of LITT treatment of mesiotemporal epilepsy (mTLE) [19,20]. A study analyzing visual deficit as a postoperative complication in patients receiving LITT treatment mTLE found that six (6.7%) reported a postoperative visual deficit. These included two homonymous hemianopsias (HHs), two quadrantanopsias, and two cranial nerve (CN) IV palsies [19]. Another study sought to identify the reason for the visual deficits after LITT by comparing four patients who developed a CN III (n = 2) or CN IV (n = 2) palsy with 22 patients who did not develop any palsies after receiving the same LITT for mTLE [20]. Their retrospective assessment of the thermal profile during ablation showed higher temperature rise along the mesial temporal lobe tissue border in affected CNs than unaffected CNs after controlling for the distance between the cranial nerves and the tissue border [20]. This finding could explain why the patient presented in this case developed transient right cranial IV palsy. Therefore, the distance between patients' cranial nerves and the site of ablation as well as the temperature of the laser should be considered when receiving LITT, potentially minimizing postoperative visual deficits.

## Conclusions

Tumors of the pineal region lie in a delicate area surrounded by vital brain structures. We report on the use of an infratentorial approach to safely ablate a pineal region glioma with minimal complications. Upon robust review of the published literature, this has not been previously described in the literature. The lack of a safe supratentorial corridor should not discourage the utilization of LITT if a safe infratentorial trajectory can be utilized. Postoperative complications included temporary right CN IV palsy potentially due to heat damage. Therefore, cranial nerve distance from the ablation site and the temperature of the laser should be considered in the preoperative planning. Further case series will be needed to ensure this approach is safe and efficacious.
